# Deviating from the Beaten Track: New Twists in Brassinosteroid Receptor Function

**DOI:** 10.3390/ijms21051561

**Published:** 2020-02-25

**Authors:** Sebastian Wolf

**Affiliations:** Centre for Organismal Studies (COS) Heidelberg, INF230, 69120 Heidelberg, Germany; sebastian.wolf@cos.uni-heidelberg.de

**Keywords:** brassinosteroids, signal transduction, signaling integration, crosstalk, receptors

## Abstract

A key feature of plants is their plastic development tailored to the environmental conditions. To integrate environmental signals with genetic growth regulatory programs, plants rely on a number of hormonal pathways, which are intimately connected at multiple levels. Brassinosteroids (BRs), a class of plant sterol hormones, are perceived by cell surface receptors and trigger responses instrumental in tailoring developmental programs to environmental cues. Arguably, BR signalling is one of the best-characterized plant signalling pathways, and the molecular composition of the core signal transduction cascade seems clear. However, BR research continues to reveal new twists to re-shape our view on this key signalling circuit. Here, exciting novel findings pointing to the plasma membrane as a key site for BR signalling modulation and integration with other pathways are reviewed and new inputs into the BR signalling pathway and emerging “non-canonical” functions of the BR receptor complex are highlighted. Together, this new evidence underscores the complexity of plant signalling integration and serves as a reminder that highly-interconnected signalling pathways frequently comprise non-linear aspects which are difficult to convey in classical conceptual models.

## 1. Introduction

Brassinosteroids (BRs) are polyhydroxylated plant sterol hormones perceived on the extracellular side of the plasma membrane by transmembrane receptors that play a central role in regulating growth and development adapted to the environment. A diverse set of forward genetics, protein–protein interaction studies, and phosphoproteomic approaches has allowed the reconstruction of an architectural model of the pathway from the plasma membrane receptors to transcription factors in the nucleus, modulating BR-responsive gene expression. This canonical model fosters the perception of BR signalling as being essentially “linear”. However, recent findings paint a considerably more complex picture, due to various types of interactions with other signalling pathways, lateral and ligand-independent inputs, and surprisingly elaborate receptor interaction dynamics at the plasma membrane. In addition to this emerging complexity of the pathway wiring, BR signalling was shown to have diverse consequences based on cell type and to have both cell-autonomous and non-cell-autonomous effects. This suggests significant modification of molecular circuitry and/or target spectrum across cell types and tissues, deciphering of which is complicated by the intractability of the tissue-level distribution of the hormone itself [1,2,3,4].

This review focuses on recent findings highlighting the non-linear aspects of BR signalling with a focus on the interplay of receptor proteins at the plasma membrane. For a detailed overview of BR functions, several excellent recent articles are available [5,6,7,8].

## 2. BR Perception and Signalling

**Figure 1:**
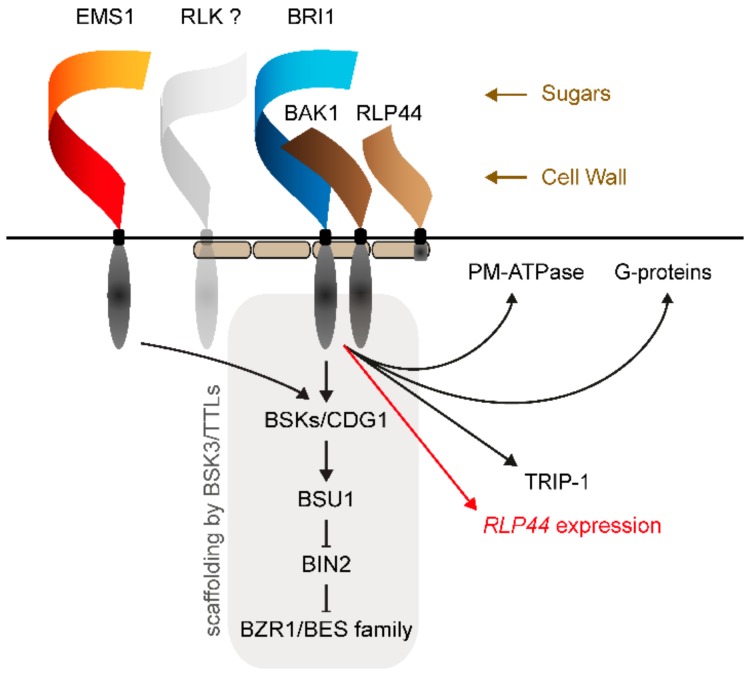
Schematic overview of the brassinosteroid (BR) signalling pathway highlighting newly discovered inputs and outputs. BRASSINOSTEROID INSENSITIVE1 (BRI1) and BRI1-ASSOCIATED KINASE1 (BAK1) form the core BR receptor complex, which forms upon ligand binding and initiates the canonical BR signalling pathway by phosphorylating BRASSINOSTEROID-SIGNALLING KINASES or CONSTITUTIVE DIFFERENTIAL GROWTH1 (BSKs/CDG1), which, in turn, activate the phosphatase BRI1 SUPPRESSOR1 (BSU1). BSU1 deactivates BRASSINOSTEROID INSENSITIVE2 (BIN2), which in the absence of BR phosphorylates BR-responsive transcription factors to prevent their nuclear translocation. Activity of the receptor complex can be influenced by sugar levels, through an unknown mechanism, and by cell wall state through RECEPTOR-LIKE PROTEIN44 (RLP44), which promotes interaction of receptor and co-receptor. The canonical BR signalling pathway can also be activated by EXCESS MICROSPOROCYTES1 (EMS1), which seems to precede BRI1 in the plant lineage, suggesting that responsiveness to BRs was acquired by evolution of BR binding in the BRI1 extracellular domain and co-option of the pre-existing signal transduction cascade. Besides the canonical BR signalling pathway, BRI1 also acts through the indicated outputs (plasma membrane (PM)-ATPase, G-proteins, TGF-β receptor interacting protein/eIF3 eukaryotic translation initiation factor subunit homolog (TRIP-1), and BR signalling-independent control of *RLP44* expression), demonstrating the complexity of BRI1-mediated signalling. Scaffolding and pathway component concentration by TETRATRICOPEPTIDE-REPEAT THIOREDOXIN-LIKE proteins (TTLs) and BSK3 is indicated as well as receptor clustering and plasma membrane subcompartmentalization by remorin proteins (rounded rectangles). Potential BRI1-RLK interactors are depicted, whereas the BR and TPD1 ligands are omitted for clarity. Black arrows indicate protein–protein interaction and/or post-translational modification, red arrow indicates transcriptional regulation, brown arrows indicate control through unknown mechanism.

BR perception is mediated by members of the largest group of plant cell surface receptors, leucine-rich-repeat receptor-like kinases (LRR-RLK), which perceive a wide variety of essential developmental and immune signalling-related cues. LRR-RLKs are characterized by an extracellular domain containing leucine-rich repeat motifs that mediate protein-protein interactions, a single-pass transmembrane domain, and a kinase domain related to mammalian Interleukin-1 receptor-associated/PELLE kinases [9,10]. BR signalling is initiated when a brassinosteroid ligand binds to the island domain of the brassinosteroid receptor BRASSINOSTEROID INSENSITIVE1 (BRI1) or its paralogues BRI1-LIKE1 (BRL1) and BRI1-LIKE3 (BRL3) [11,12,13,14,15], which creates a binding interface with for co-receptors of the SOMATIC EMBRYOGENESIS RECEPTOR-LIKE KINASE (SERK) class such as BRI1-ASSOCIATED KINASE1 (BAK1)/SERK3 [16,17,18,19]. Heterodimer formation between receptor and co-receptor then leads to juxtaposition of the intracellular kinase domains, initiating trans- and autophosphorylation [20,21,22], as well as the phosphorylation and dissociation of inhibitory factors [23,24,25]. Phosphorylation of the kinase domains of the BR receptor complex leads to an increase in kinase activity and the creation of binding interfaces with downstream signalling components, resulting in phosphorylation of the latter. These immediate downstream components are proteins of the class of receptor-like cytoplasmic kinases [26,27,28], which, in turn, phosphorylate and activate the phosphatase BRI1 SUPPRESSOR1 (BSU1) [28,29,30]. BSU1 dephosphorylates and thereby inactivates the negative regulator BRASSINOSTEROID INSENSITIVE2 (BIN2), a member of the glycogen synthase kinase 3 (GSK3) class of kinases [31]. In the absence of BRs, BIN2 phosphorylates BR-responsive transcription factors such as BRASSINAZOLE-RESISTANT1 (BZR1) and BRI1-EMS-SUPPRESSOR1 (BES1) [32,33,34,35,36]. When BR perception leads to the inactivation and degradation of BIN2 [28,37,38], the transcription factors can be dephosphorylated and released from cytosolic sequestration mediated by 14-3-3 proteins and travel to the nucleus, where they mediate BR-responsive gene expression [39,40,41] (Figure 1). BES1 and BZR1 have been identified through gain-of-function alleles that render plants partially resistant towards inhibition of BR biosynthesis, demonstrating that these transcription factors can regulate BR-responsive genes [34,35]. Loss-of-function mutants of BES1 and BZR1 are indistinguishable from wild type plants, which was interpreted as evidence of redundancy. Recently, this was confirmed by studies analysing mutants in multiple transcription factors of the BZR1/BES1 family, providing long-missing loss-of-function evidence for a central role of BZR1 and BES1 in BR signalling [42,43]. Indeed, higher order BZR1/BES1 family mutants show gross morphological phenotypes very similar to *bri1 brl1 brl3* [44], and are not responsive to BR treatment with respect to gene expression [42]. Thus, it seems that the basic architecture of the BR signalling pathway is reasonably well understood. However, the complexity of how BR signalling intersects with other pathways to control growth and stress responses is only beginning to emerge.

## 3. BR Signalling Is Intimately Connected to Other Growth-Regulatory and Stress-Response Pathways

It has long been known that BR signalling shows cross-talk with other hormonal and stress-response pathways [45,46,47,48]. The interaction with auxin, arguably the most-studied example, is particularly instructive in demonstrating how intricate the wiring between signalling pathways can be. Importantly, the BR and auxin pathways are interdigitating at multiple levels of the signalling cascade, hormone biosynthesis, and the target genes [49,50,51,52,53]. For example, BIN2 interacts with and phosphorylates AUXIN RESPONSE FACTORS (ARFs) to alter their activity [54,55]. The two pathways also converge on the promoters of some common target genes [56,57,58,59] and BZR1 and ARF6 physically interact [59]. Moreover, auxin transcriptionally regulates BR biosynthesis and signalling genes [60,61,62,63], and can prevent nuclear accumulation of BR-responsive transcription factors [52], whereas BRs regulate auxin signalling and transport [44,58,64,65,66,67]. Adding to this already diverse set of interactions between the two pathways, another mechanism of auxin–BR crosstalk is described in a recent preprint [68]: the authors report that BR signalling negatively regulates PIN-like (PIL) auxin transport facilitators at the ER membrane [69]. As auxin is sequestered in the ER to modulate signalling [70,71,72,73,74,75], reduction of PILs abundance increases the concentration of auxin in the nucleus, promoting auxin signalling. Thus, BR signalling not only regulates auxin responses and intercellular transport, but also nuclear auxin levels [68]. 

Similar to what is observed with auxin, evidence for the intertwining of BR signalling with several other pathways in a complex and diverse manner is accumulating, whereby two particularly prominent integration “hotspots” have emerged. First, BIN2 and its paralogues connect BR signalling to root hair patterning [76], stomatal development [77,78,79], ABSCISIC ACID (ABA) signalling [80,81,82,83], and other pathways [55,84]. Second, BR-responsive transcription factors such as BES1 and BZR1 interact with a diverse set of other factors to alter their DNA-binding characteristics and the target spectrum of the transcriptional response [85,86,87,88,89,90,91,92,93]. Moreover, protein abundance of BR-responsive transcription factors is influenced by autophagy [94], light [95], and strigolactone signalling, which connects BR signalling to the control of shoot branching imparted by the interaction of the transcription factors with MORE AXILLARY BRANCHES2 (MAX2), an F-box protein that promotes degradation in the proteasome [96]. In addition to this cross talk mediated by BIN2 and the BR-responsive transcription factors, recent work has also uncovered branching and integration points of BR signalling at the level of the plasma membrane, through which the BR receptor complex emerged as a key site for regulation of BR signalling strength, as well as signalling integration and outbranching.

## 4. Modulation of BR Signalling at the Plasma Membrane 

### 4.1. Control of Receptor Abundance

It has been established that BR ligand perception triggering kinase signalling occurs at the plasma membrane [97]. Thus, an obvious way in which BR signalling strength is modulated is through controlling the amount of the receptor BRI1. In line with this, the BR signalling response is very sensitive to reduced BRI1 abundance at the plasma membrane [98,99,100]. Conversely, increased receptor amounts lead to hyper-responsiveness to external application of BRs and increased resistance to depletion of endogenous BRs [101,102,103]. The amount of BRI1 at the plasma membrane is broadly controlled by the rate of synthesis, passage through ER quality control, secretion, and endocytosis. Recently, a number of studies have provided valuable insight into the mechanism of BRI1 removal from the plasma membrane. BRI1 and its co-receptors undergo endocytosis in a clathrin-dependent manner—as expected to occur for most transmembrane receptors [101,104,105,106,107,108]. Experiments with fluorescently-labelled castasterone (a bioactive BR) have shown that BRI1 and its BR ligands are taken up together and eventually delivered to the vacuole for degradation [97]. Modification of the BRI1 cytosolic domain with K63-linked ubiquitin chains acts as a dual signal for endocytic uptake and endosomal sorting towards the vacuole [109]. Stabilization of BRI1 at the plasma membrane, either by general inhibition of endocytosis or by interfering with ubiquitination, increases BR signalling strength, whereas increased uptake through translational fusion with ubiquitin attenuates BR responses, confirming that the plasma membrane is the relevant compartment for BRI1 signalling [97,109]. Interestingly, abundance of BRI1 at the plasma membrane is also affected by feedback from the cytoskeleton through the microtubule-associated protein CLASP1 [100], whereas actin rearrangements trigger BR signalling activation through an unknown mechanism [65].

### 4.2. Sub-compartmentalization and Clustering

Over the course of the last few years it has become more and more apparent that most, if not all, plant transmembrane proteins are not distributed uniformly in the plane of the plasma membrane, but typically localize to domains in size <1 µM that are marked by flotillins (FLOT) and the plant-specific remorin (REM) class of membrane-associated proteins [110,111,112,113]. It is assumed that these protein classes are involved in the generation and the maintenance of membrane nanodomains, of which several types co-exist in a given cell [114], by way of their capability to oligomerize and provide a scaffold for the clustering of transmembrane domain proteins such as RLKs [115,116,117,118,119]. The cell wall also contributes to the size and dynamics of nanodomains [120], potentially by restricting lateral mobility of plant transmembrane proteins [121,122]. Alternatively, receptor clustering after cell wall alterations could be indicative of a cellular response to mechanical stimuli [120].

Interestingly, remorin proteins can also directly affect signalling as shown by a recent study in rice [123]. OsREM4.1, which is upregulated by ABA signalling, interacts with rice SERK1 and thus prevents complex formation with OsBRI1 unless it is phosphorylated by the latter. Consistent with this, *OsREM4.1*-overexpressing plants are dwarfed and resistant to BR treatment, whereas RNAi plants showed phenotypes consistent with increased BR response. Thus, OsREM4.1 function seems to go beyond scaffolding by directly interfering with BR receptor complex formation [123].

More generally, segregation of receptors in the relatively immobile nanodomains is assumed to restrict the access to potential interaction partners and thus allow tighter control of signalling activation. Indeed, it has been recently demonstrated that BRI1 resides in nanoclusters that are marked by FLOT1 and REM6.2 [120,124,125,126]. BRI1 is spatially separated from FLS2, which is present in nanodomains marked by REM1.2 and REM1.3, despite the fact that both receptors depend on SERK co-receptors such as BAK1 [124]. The relatively immobile BRI1 nanoclusters are further stabilized by BR application, whereas depletion of BRs increases BRI1 mobility, suggesting that increased activity is associated with clustering. This is in line with recent findings in the broader field of receptor-mediated signalling, pointing towards receptor clustering [127,128] or even the formation of “biomolecular condensates” [129] to enhance the recruitment and activity of downstream signalling components and provide a noise filtering mechanism to prevent spurious activation and allow a graded response [130,131,132,133]. This general theme of clustering and concentration of receptors and their downstream targets is supported by two additional studies. BRASSINOSTEROID SIGNALLING KINSASE3 (BSK3) was recently shown to act as a scaffold for other BR signalling components [134]. BSK3 is membrane-associated through N-terminal myristoylation and directly interacts with BRI1 and BSU1, consistent with the described role of BSKs as intermediary signalling component downstream of BRI1 [26,27]. However, BSK3 also forms homodimers, heterodimers with BSK1, and interacts with BIN2, which is one step removed in the signalling cascade. BSK3 kinase activity is at least partially dispensable, suggesting that BSK3, mutants of which show reduced BR sensitivity [26], primarily acts as a scaffolding protein tethering BR signalling components to the plasma membrane [134]. In line with this, a recent study identified TETRATRICOPEPTIDE-REPEAT THIOREDOXIN-LIKE proteins (TTLs), which combine several protein interaction motifs, i.e. have the potential for multivalency (engaging in several simultaneous protein–protein interactions), as positive regulators of BR signalling by scaffolding BR signalling components [135]. TTL proteins become membrane-associated when BR signalling is activated and interact with BRI1, BSU1, BSK1, BIN2, and the BZR1 transcription factor, tethering many of the required signalling components in multiprotein complexes [135]. Thus, rather than as a series of isolated reactions, BR signalling might be better thought of as a signalling assembly, which could serve as a mechanism ensuring signalling specificity in light of the diverse roles that several of the signalling components engage in. Interestingly, BIN2 has recently been shown to be part of another protein complex organized by scaffolding, which links BR inputs to stomatal development [77,78,136].

What are the functional consequences of clustering for receptor-mediated signalling? Clustering is thought to facilitate and fine-tune effective signalling by sequestering components away from unfavourable interactions, by amplifying and filtering the signal against noise (spurious activation), and by providing a buffer against concentration differences of the interaction partners. It remains to be shown whether clustering of receptors and downstream components in and close to the plasma membrane thus constitutes a case of true liquid–liquid phase separation or liquid unmixing [137,138], what constitutes the mechanistic functional benefit in case of BR signalling, and how widespread these mechanisms are for LRR-RLK-mediated signalling in general.

## 5. New Inputs and Outputs for BRI1 Signalling

In addition to clustering and possible condensation, outbranching and divergence from the linear BR signalling pathway has also been observed at the plasma membrane. For example, BRI1 enhances PM-ATPase activity within hours through the canonical BR signalling pathway, via transcriptional upregulation of SMALL AUXIN UPREGULATED (SAUR) genes [139]. In addition, a faster PM-ATPase-dependent response to BRs leading to cell wall expansion and membrane hyperpolarization was observed [140]. These effects were suggested to be caused by direct regulation of PM-ATPase activity by BRI1 through phosphorylation, constituting a lateral output of BRI1 activity independent of downstream BR signalling. This is reminiscent of an earlier study, which revealed that BRI1 phosphorylates a homolog of the mammalian TGF-β receptor interacting protein/eIF3 eukaryotic translation initiation factor subunit (TRIP-1) [141]. However, the function of TRIP-1 and whether it bypasses canonical BR signalling is not clear at present state.

Multiple links exist between sugar signalling [142] and the BR signalling pathway [143,144,145]. For example, sugar availability feeds back on BZR1 abundance through TARGET OF RAPAMYCIN (TOR) signalling [146]. Thus, TOR integrates carbon availability by gating BR signalling strength through regulating the abundance of BR-responsive transcription factors. However, sugar signalling is also directly linked to the BR receptor complex [147]. The interaction of BRI1 and BAK1 was shown to be influenced by glucose levels within 24 hours of application. In addition, the BR receptors interact with and phosphorylate G-protein subunits to control sugar-responsive growth. Hence, sugar seems to provide an additional input into the BR signalling pathway, modulating complex formation, whereas regulation of G-proteins represents another branching point at the level of the BR receptors [147].

One of the primary outputs of BR signalling is altering cell wall properties to control elongation growth. This is reflected in the abundance of cell-wall-related BR target genes [58,148,149], but also in post-translational control of enzymes controlling extracellular pH [140] and cell wall biosynthesis [150]. In addition, BR signalling might exert control over cell wall properties through regulation of microtubule alignment as a determining factor of cellulose and therefore cell wall mechanical anisotropy [151,152]. However, the cell wall also feeds back on BR signalling [153]. The state of pectin, an important component of the plant cell wall and a dynamic regulator of its properties [154,155,156,157], is relayed to the BR receptor complex by RECEPTOR-LIKE PROTEIN44 (RLP44), which directly interacts with both BRI1 and BAK1, likely acting as a scaffold to promote their association [103,158,159]. In line with this notion, RLP44 is sufficient to promote BR signalling, at least partially independent of BRs [103]. 

RLP44 is also at the centre of yet another unexpected deviation from canonical BR signalling. In addition to promoting BR signalling in response to cell wall cues, RLP44 is involved in the control of vascular cell fate through the promotion of phytosulfokine (PSK) signalling [158]. The two main PSK receptors are close relatives to BRI1 within family X of LRR-RLKs and share with BRI1 the requirement for SERK co-receptors. RLP44 promotes PSK signalling, which has been linked to BR signalling before [160,161], through the same mechanisms as described for the BR receptor complex⁠—by acting as a scaffold to foster interaction between receptor and co-receptor. Strikingly, this promotion of PSK signalling is dependent on the presence of BRI1, but not on an intact downstream BR signalling pathway. This is explained by BRI1-dependent, but BR signalling-independent control of *RLP44* expression. Thus, BRI1 can control gene expression through an alternative, as of yet unidentified pathway [158].

## 6. Signalling Integration at the Plasma Membrane

LRR-RLKs interactions have emerged to be more sophisticated than the interaction between ligand-binding receptors and their co-receptors, with diverse outcomes for the modulation of signalling strength. For example, BAK1-interacting receptor-like kinases (BIRs) [162] interact with SERKs but have mostly negative effects on SERK function. BIR1, 2, and 3 were shown to negatively regulate BAK1-dependent immune signalling [162,163,164], whereas BIR3 interaction with BAK1 also prevents complex formation of the latter with BRI1, thus negatively regulating BR signalling [164,165]. In the presence of BRs, BIR3 is released from SERKs and BRI1 [164].

A recent study has provided a fresh view on LRR-RLKs, suggesting that their interactions at the plasma membrane are a lot more complex than previously considered [166]. The study is based on recombinant expression of 200 LRR-RLK extracellular domains in insect cells and testing of their interactions in an enhanced all-by-all pairwise interaction screen. Notably, the screen occurs in the absence of ligands, which are believed to be required as “molecular glue” to enable heteromeric interaction between ligand-binding receptors and their co-receptors [167]. The screen employed by Smakowska-Lukan et al., previously developed for Drosophila extracellular proteins [168], uses oligomerized recombinant proteins to enhance avidity and identify candidate interactors based on the assumption that even in the absence of ligands, a basal level of affinity remains between proteins that engage in heterodimerization in the plasma membrane. Moreover, ligand-independent interactions between LRR-RLK and preformed receptor–co-receptor complexes have been described [169,170]. Of the 40,000 potential interactions with 200 extracellular domains tested, 567 were supported by robust evidence and classified as high confidence interactions. Supporting the validity of the interaction network, the intracellular kinase domains of proteins that showed extracellular domain interactions were also more likely to interact in a yeast 2-hybrid assay than randomly selected LRR-RLK kinase domains. Despite the absence of ligands and the removal of the extracellular domains from the context of the plasma membrane, the retrieval of known interactions, such as between BRI1 and BAK1 and between FLAGELLIN SENSITIVE2 (FLS2) and BAK1, the latter widely assumed to be strictly ligand-dependent, validate the approach. The interaction matrix yielded a number of interesting hypotheses concerning the emerging LRR-RLK network, with the caveat that network structure in planta might be greatly modified through tissue-specific expression and membrane sub-compartmentalization [112,124,125,126]. Smakowska-Lukan et al. revealed that individual LRR-RLKs differed widely in the number of interaction partners. Smaller LRR-RLK proteins such as BAK1 and APEX had the largest number of interactions and were critical for network integrity, suggesting that they are involved in maintaining adequate signalling outputs even of those receptors that are several steps removed in the network. Thus, signalling outputs by individual LRR-RLKs embedded in this interaction network seem to be fine-tuned by direct interaction partners as well as by proteins with indirect connections. In agreement with the notion of a perception network, rather than isolated, singular receptors, the study also revealed several new LRR-RLKs that might affect BR signalling by interacting with BRI1. When the authors tested BR-responsive growth of mutants with lesions in potential BRI1 interactors obtained from the in vitro interaction screen, mutants in seven of eight high confidence BRI1 interactors showed reduced hypocotyl elongation in response to BR, whereas this read-out was affected in only one of seven mutants in candidates without evidence for interaction. Importantly, the high confidence interactors included the known BR signalling components BAK1 and SERK4, but also potential novel players such as RECEPTOR-LIKE PROTEIN KINASE1 (RPK1), HAESA-LIKE2 (HSL2), and BARELY ANY MERISTEM3 (BAM3), which were not identified by a previous plasma membrane interaction screen [171]. At the very least, this shows that BRI1 engages with more partners than previously thought, underscoring the complexity of plasma membrane receptor dynamics. Future work should address how these potential novel interaction partners exert their influence on BR signalling mechanistically and how the interaction network can be integrated in an updated model of BR signalling. For example, the complexity of interactions revealed in the interactome analysis as well as in several other recent studies suggests that there is competition for binding between LRR-RLKs, modified by the presence of extracellular ligands and LRR-RLPs [158,165,172,173,174]. 

One has to wonder how this complex network is further elaborated through the presence of RLPs, which were not included in the interaction screen by Smakowska-Lukan et al. For example, TOO MANY MOUTHS (TMM) or RLP17 was shown to affect specificity of ERECTA family RLKs for binding of EPIDERMAL PATTERNING FACTOR (EPF) peptides [172], whereas the important meristem regulator CLAVATA2 (RLP10) was suggested to be essential for the function of CLAVATA3/ESR-RELATED (CLE) peptide-binding RLKs [175]. Another recent example is provided by competition of BRI1 and PHYTOSULFOKINE RECEPTOR1 (PSKR1) for RLP44. As RLP44 is required for normal PSKR1 function, increased interaction of BRI1 with RLP44 leads to PSK signalling deficiency [173]. 

## 7. Extracellular Diversity and Intracellular Conservation

As illustrated above, the interaction dynamics of LRR-RLK such as BRI1, driven by the extracellular LRR domains, has emerged as an important mechanism mediating integration and fine-tuning of signalling. Downstream signalling, on the other hand, seems considerably more conserved and depends on the intracellular kinase domain. Recent studies have helped to develop a model of LRR-RLK signalling evolution, according to which the acquisition of novel signalling molecules by rapid diversification of RLK-LRK extracellular domains can be plugged onto existing signal transduction cascades to diversify organismal responses to the environment. 

A prediction following from the current model of LRR-RLK signalling is that effects downstream of the plasma membrane, for example changes in gene expression, are exclusively mediated by the intracellular kinase domain. Consistent with this idea, chimeric receptors respond to ligands specific for their extracellular domain, but enact responses depending on the kinase domain [176,177]. For example, a chimeric protein consisting of the BRI1 extracellular domain and the HAESA (HAE) kinase domain complemented the abscission defect of *hae hsl2* double mutant when expressed from the *HAE* promoter. However, complementation was abolished when the BR binding site of the BRI1 extracellular domain was mutated, suggesting that BR-induced heterodimerization with SERK co-receptors is required for the chimeric receptor to regulate abscission through the HAE signalling pathway [178]. 

Underscoring this uncoupling of extracellular domain variability and kinase domain targets was provided by recent studies tackling the relationship of BRI1 and another family X LRR-RLK, EXCESS MICROSPOROCYTES1 (EMS1), also known as EXTRA SPOROGENOUS CELLS (EXS). Using SERK1 and SERK2 as co receptors, EMS1/EXS is involved in sensing of the peptide TAPETUM DETERMINANT1 (TPD1) to specify the tapetum, the cell layer nurturing the pollen grains during the last phase of their development [179,180,181,182,183]. Chen et al. [43], (in agreement with the study [42] discussed above), revealed that plants in which multiple members of the BZR1/BES1 transcription factor family are mutated, show gross morphological phenotypes very similar to those of *bri1* mutants. However, BZR1/BES1 higher order mutants also show male sterility due to impaired anther development, a phenotype that is independent of BRI1 signalling, but reminiscent of *ems1* and *tpd1* mutants. Indeed, BES1 and BZR1 gain-of-function mutants can rescue the *ems1* and *tpd1* sterility phenotype, and EMS1 signalling activates BES1 [43]. In addition, through elegant genetic experiments, Zheng et al. show that despite the diversity of their extracellular ligands, the kinase domains of BRI1 and EMS1 are interchangeable, demonstrating that they trigger the same downstream responses [184]. Chimeric proteins consisting of the BRI1 extracellular domain and the EMS1 kinase domain, expressed under control of the *BRI1* promoter, were able to rescue *bri1* phenotypes and restore BR responsiveness, whereas chimeras with other kinase domains such as that of the closely-related PSKR1, were not. Conversely, the BRI1 kinase domain was able to rescue the *ems1* mutant when fused with the EMS extracellular domain. EMS1 and TPD1 can partially rescue the *bri1-116* mutant independently of BRs when co-expressed under control of the *BRI1* promoter. In addition, *BRI1* can rescue *ems1* when expressed under the control of the *EMS1* promoter. These results also highlight the complementary nature of the *BRI1* and *EMS1* expression domains, as EMS1 under control of the *BRI1* promoter cannot rescue *ems1* [184]. Consistent with the hypothesis that BRI1 and EMS1 trigger similar downstream responses in different expression domains, *bri1 ems1* double mutants combine the effects of the single mutants and thus strongly resemble BZR1/BES1 family multiple mutants [42]. These findings maybe should rekindle interest in BRI-LIKE2 (BRL2), which is not able to interact with BRs but has a kinase domain similar to BRI1 [13,185,186]. 

BRI1 is only found in seed plants [185], whereas orthologues of EMS1 and TPD1 are present in all land plants including ferns, mosses, lycophytes, and liverworts. The biochemical function of these orthologues is conserved, as EMS1 and TPD1 of the moss *Physcomitrella patens* could rescue Arabidopsis *ems1* and *tpd1*, respectively [184]. In addition, co-expression of *Physcomitrella EMS1* and *TPD1* under control of the *BRI1* promoter rescued *bri1* mutants and the *Physcomitrella* EMS1 kinase domain was functional in a chimera with the Arabidopsis BRI1 extracellular domain. Thus, presence of EMS1 and TPD1 and a conserved downstream signalling seems to precede evolution and neofunctionalization of the BRI1 extracellular domain. This would imply that perceptiveness to novel ligands can be acquired independently of kinase domain evolution and grafted onto existing signal transduction pathways, presumably accompanied by diversification of expression domains following gene duplication events. As discussed above, a cell-type specific modulation, e.g. through interacting transcription factors, enables a diversity of outputs despite a similar pathway architecture. 

## 8. Conclusions

Brassinosteroid signalling is arguable one the best-studied plant signalling pathways, yet exciting new discoveries are reported with high frequency. It is becoming more and more clear that BR signalling is intimately connected to a number of other pathways and in light of the emerging complexity of plasma membrane receptor interaction networks, this number can be expected to further increase. In addition, recent studies have made it clear that BR receptor function exerts itself through more than one route. While canonical BR signalling, leading to large scale transcriptional rearrangements, certainly explains the bulk of the observed phenotypes, non-canonical signalling and alternative outputs play important roles in tailoring plant development to the environmental conditions. Moreover, additional inputs into the pathway and the role of modulators of receptor–co-receptor interactions remain underexplored. However, there also remain important unanswered questions related to the core pathway itself. For example, what is the mechanism by which BR signalling mediates one of its signature effects, the promotion of elongation growth? How does restructuring of the cellular wiring explain the pronounced cell type specific difference in the consequences of BR signalling? What led to the acquisition of perceptiveness to sterols and its grafting onto an existing signal transduction pathway during seed plant evolution? Thus, the coming years should pose enough challenges to keep the vibrant community of BR signalling researchers occupied and reveal even more unexpected plot twists produced by this versatile pathway.
